# Research on the Impact of Generative Artificial Intelligence Usage Behavior on the Learning Outcomes of Higher Vocational Students

**DOI:** 10.3390/bs16071166

**Published:** 2026-07-10

**Authors:** Yafeng Song, Kangjian Zhao, Li Li, Wei Dong

**Affiliations:** School of Education, Tianjin University, Tianjin 300350, China; zhaokj@tju.edu.cn (K.Z.); lili061@tju.edu.cn (L.L.)

**Keywords:** higher vocational students, generative artificial intelligence, usage behavior, perceived learning outcomes, impact

## Abstract

Generative Artificial Intelligence (GenAI) has been increasingly integrated into vocational education teaching and students’ learning. Thus, instructing higher vocational students to use GenAI effectively and improving their self-reported perceptions of learning outcomes are critical. Based on the talent development requirements of vocational education, this study developed and validated the GenAI Usage Behavior Scale and the Higher Vocational Students’ Perceived Learning Outcomes Scale. Subsequently, an empirical analysis was conducted using data from 1110 valid questionnaires collected from Chinese higher vocational students. According to the descriptive statistical analysis, the overall usage behavior of higher vocational students was generally at a medium–high level. They performed relatively well in terms of usage habits, moderately in usage contexts, and showed a relatively low frequency of use. The overall evaluation for higher vocational students’ perceived learning outcomes was rated as above average with competency development ranking highest, followed by skill application and knowledge mastery. As for group differences, the results of the independent samples *t*-test and one-way analysis of variance (ANOVA) revealed no overall significant differences in perceived learning outcomes across genders. Significant differences were only observed in the skill application dimension across grades, while all dimensions of perceived learning outcomes showed statistically significant disparities across major categories. From the perspective of the correlational mechanism, correlation and regression analyses revealed a statistically significant negative correlation between GenAI usage frequency and perceived learning outcomes. In contrast, usage habits and contexts exhibited statistically significant positive correlations. Among these factors, usage habits demonstrated stronger explanatory power. Accordingly, colleges, teachers, and students each must have clear priorities and work collaboratively to enhance students’ ability to use GenAI effectively, thereby leveraging its supportive role in improving perceived learning outcomes for higher vocational students more efficiently.

## 1. Introduction

At present, the Fourth Industrial Revolution is deepening, with emerging technologies such as artificial intelligence, big data, and the Internet of Things, reshaping social production, economic structures, and educational paradigms (Oke & Fernandes, 2020). In the field of education, GenAI tools such as ChatGPT, Gemini and Kimi have robust capabilities in data processing, intelligent interaction, and creative generation, which have transformed traditional approaches to knowledge acquisition, enhanced the efficiency of knowledge production, and shown particular promise in facilitating personalized learning (Kasneci et al., 2023; Baidoo-Anu & Owusu Ansah, 2023). Thus, the widespread application of emerging technologies is driving the transformation of the education system toward Education 4.0. The World Economic Forum defines the “Education 4.0 Framework” as teaching that adapts learning content and experiences to future demands. In 2024, it released the report *Shaping the Future of Learning: The Role of AI in Education 4.0*, which elaborates on four core directions of AI-enabled education: supporting teachers, optimizing assessment, enhancing digital literacy, and delivering personalized learning experiences (World Economic Forum, 2024).

As the type of education most closely aligned with industrial demands, the emergence of the Vocational Education 4.0 concept is attributed to the accelerated advancement of Industry 4.0. Originating in Germany, Vocational Education 4.0 takes advancing digital construction, reform, and innovation within vocational education as its core tenet. To address this trend, the United Nations Educational, Scientific and Cultural Organization (UNESCO) analyzed the positioning, roles, and implementation approaches of higher education across nations, based on AI strategy documents from 56 countries, and proposed integrating AI competency development into all disciplinary domains (United Nations Educational, Scientific and Cultural Organization, 2025). In April of the same year, the European Commission explicitly proposed establishing AI Skills Academies and launching AI apprenticeship programs to develop AI-skilled professionals through real-world industry projects (European Commission, 2025). Subsequently, the Organization for Economic Co-operation and Development (OECD) focused on the transformative potential of innovative technologies in vocational education and training, particularly GenAI. It examined how these technologies could reshape learning environments, experiences, and outcomes, drawing on practical cases from member states including the United Kingdom and Switzerland (Organisation for Economic Co-Operation and Development, 2025).

However, the introduction of innovative technological tools provides only a necessary foundation. A key factor influencing GenAI effectiveness in Vocational Education 4.0 is students’ AI usage behavior. *The AI Competency Framework for Students*, released by UNESCO in 2024, offers authoritative and theoretical guidance. It divides students’ GenAI usage behavior standards into four core dimensions: human-oriented thinking, AI ethics, AI technology and application, and AI system design, each progressively structured from understanding to application and creation (United Nations Educational, Scientific and Cultural Organization, 2024). Relevant research indicates that GenAI usage has a significant positive impact on students’ learning outcomes and provides personalized, interactive learning experiences (Topaz et al., 2025). GenAI’s practical applications include assisting with digital multimodal writing, stimulating brainstorming processes, facilitating data visualization and information integration, etc. (M. Liu et al., 2024; C. K. Y. Chan & Hu, 2023). Existing research has also found differences in learning outcomes among different levels of GenAI usage behavior. Taking AI-assisted writing as an example, students with higher-level usage behavior are able to formulate descriptive and context-aware prompts, whereas those with lower-level usage behavior tend to adopt general prompts. Ultimately, students at higher levels achieve better learning outcomes (J. Kim et al., 2026).

Most existing studies focus on undergraduate students, reflecting insufficient attention to the vocational education population. As the core group of vocational education, higher vocational students differ distinctly from undergraduates in terms of learning traits and training objectives (Billett, 2011). Accordingly, the mechanisms and practical manifestations of GenAI use on perceived learning outcomes are distinctive, and relevant research conclusions targeting undergraduates cannot be directly generalized to this group.

This study used higher vocational students as research subjects. Drawing on the literature review and aligned with the inherent features of vocational education, scientifically sound and targeted scales were developed to measure GenAI usage behavior and higher vocational students’ perceived learning outcomes. Through statistical analysis of questionnaire data, the following questions were addressed:

1. What is the core connotation of GenAI usage behavior?
2. What are the components of higher vocational students’ perceived learning outcomes?
3. Which dimensions of generative AI usage behavior exhibit significant correlations with vocational college students’ perceived learning outcomes? If such correlations exist, what are their directional patterns?
4. How can learning outcomes among higher vocational students be enhanced by optimizing their GenAI usage behavior?

## 2. Literature Review

### 2.1. Research on GenAI Usage Behavior

GenAI tools such as ChatGPT, Gemini, Claude and others have been widely integrated into work, study, and daily life. The origin of GenAI dates back to 2017, when Google’s research team proposed the transformer architecture in the paper *Attention Is All You Need*, laying a solid foundation for the subsequent evolution of generative models (Vaswani et al., 2017). Since the official launch of ChatGPT in late 2022, GenAI has entered mainstream public awareness and sparked a global surge in AI technological development. A host of conversational large language models (LLMs) have since emerged. GenAI usage contexts now cover content creation, software development, customer service, education, scientific research and many other fields (Dwivedi et al., 2023). In addition to greatly boosting work efficiency, the popularization of GenAI is profoundly reshaping the basic paradigms of information generation, dissemination, and application (Bommasani et al., 2022).

To clarify the core meaning of GenAI usage behavior, the concept of GenAI must first be defined. As a subset of artificial intelligence, GenAI refers to multimodal machine learning technology that generates new content including texts, images, and videos via specific algorithmic models (Feuerriegel et al., 2024). Its emergence signifies a paradigm shift in AI technology from traditional analytical AI to GenAI. Thus, GenAI usage behavior cannot be fully captured by the traditional definition of digital literacy. Traditional digital literacy generally refers to an individual’s ability to acquire, understand, manage, and exchange information in traditional information technology environments, as well as to solve practical problems and participate in social interactions with digital tools. It emphasizes information acquisition, storage, management, and basic operational skills (Martin, 2006). However, GenAI usage behavior is embedded in the GenAI technological context, emphasizing users’ effective interaction with generative models, their capacity to evaluate the quality of AI-generated content, and their competence to integrate and apply such outputs (Tlili et al., 2023). Studies have put forward multiple literacy concepts closely related to GenAI applications, such as AI literacy, prompt literacy, algorithmic literacy, and data literacy (Long & Magerko, 2020; Zamfirescu-Pereira et al., 2023; Gran et al., 2021; Pangrazio & Selwyn, 2019).

AI literacy is a broad concept that mainly covers the ability to perceive, utilize, and critically evaluate AI technologies. It encompasses the knowledge of basic AI concepts and technical principles, familiarity with practical usage contexts, as well as human-oriented ethical awareness and social responsibility (Ng et al., 2021). Closely linked to GenAI usage behavior, prompt literacy denotes users’ capacity to design, optimize, and iterate prompts to steer AI toward generating targeted outputs (White et al., 2023). Algorithmic literacy emphasizes on the understanding of the operational logic of algorithmic systems, including how algorithms filter, rank, and recommend information and further shape information exposure (Dogruel et al., 2022). Data literacy refers to foundational competencies in data collection, processing, interpretation, and practical application (Wolff et al., 2016). All the above literacies differ in their core focuses. GenAI usage behavior requires the integration of these literacy dimensions into a competency framework tailored to generative AI technologies.

Given the rapid development of GenAI technology and a focus on Chinese higher vocational education, scholars have increasingly turned their attention to GenAI’s application in this field. They systematically examined the current status of higher vocational students’ GenAI literacy and its influencing factors. Research indicates that while students generally hold a positive attitude toward GenAI, there are significant differences in their practical application competency in this technology. Students’ performance is comparatively weaker in core competency dimensions, including prompt design, output evaluation, and ethical reasoning (Song et al., 2026). Some studies show that higher vocational students use GenAI as an auxiliary tool to complete specific tasks; however, their usage behavior remains superficial, lacking systematic and critical awareness of its application (Zou et al., 2025). Overall, research on GenAI literacy in Chinese higher vocational education has entered a phase of rapid development; however, authoritative criteria for tools measuring GenAI usage behavior remain lacking. Therefore, an analysis framework and measurement tools tailored to both Chinese higher vocational education and general higher education contexts must be developed and designed to meet the new talent cultivation requirements brought about by digital transformation.

Accordingly, GenAI usage behavior is defined as an integrated competency that enables users to: (1) apply GenAI tools rationally in daily study and life; (2) identify, filter, synthesize, and apply content generated by GenAI; (3) utilize such tools to fulfill diverse demands including information searching, learning assistance and vocational skill development; (4) promote the improvement of knowledge mastery, skills application and sustainable development competence.

### 2.2. Research on the Perceived Learning Outcomes of Higher Vocational Students

Higher vocational education is employment-oriented, with a core mission of cultivating high-quality technical and skilled talents. Compared to undergraduate education, which prioritizes academic exam scores as learning outcomes, higher vocational education’s assessment framework places greater emphasis on students’ skill proficiency and occupational adaptability. In this context, exam scores are not the sole criterion for assessing learning outcomes (Richardson et al., 2012; Lucas et al., 2012). In a narrow sense, higher vocational students’ learning outcomes are primarily their assessment scores in course study and mastery of professional skills (Lucas et al., 2012; Wheelahan, 2015). In a broad sense, the connotation is much richer, encompassing not only professional knowledge and technical skills but also professional ethics and the craftsmanship spirit (De Bruijn & Leeman, 2011), team collaboration ability (Zou et al., 2017), and job adaptability (Z. Bai et al., 2025). In the context of overlapping challenges like the technological revolution and green transition, *UNESCO’s Strategy for Technical and Vocational Education and Training* (TVET), 2022–2029, emphasizes that TVET should cultivate students’ future-oriented, high-level literacy and transferable competencies, such as digital literacy, green skills, STEM capabilities, and critical thinking, rather than solely focusing on enhancing their job-specific skills (United Nations Educational, Scientific and Cultural Organization, 2022). The training objective of vocational education has shifted from shaping proficient operators for specific positions to cultivating lifelong learners who can continuously adapt to technological changes and industrial upgrading. Drawing on the basic framework for training objectives of vocational education and UNESCO’s updated requirements for higher vocational students, the learning outcomes of higher vocational students are defined as comprehensive achievements acquired through educational activities, including in-class study, practical training, and internships. These perceived outcomes are divided into three core dimensions: knowledge mastery, skill application and competency development.

### 2.3. Research on the Relationship Between GenAI Usage and Perceived Learning Outcomes

Existing research concludes that the factors influencing students’ perceived learning outcomes include internal and external factors. Internal factors are individual-related, including intelligence (Cui, 2011), learning engagement (Lei et al., 2018), self-efficacy (Honicke & Broadbent, 2016), learning-related emotions (Hayat et al., 2020), and learning motivation (J. Li & Xue, 2023). External factors encompass school climate (R. C. H. Chan & Lam, 2023), family environment (J. Liu et al., 2020), teaching behavior (Baber, 2020), peer interaction (Qureshi et al., 2023), and the use of learning tools (Iqbal et al., 2025). Along with the rapid development of AI technology, GenAI has gradually become a widely adopted assistive learning tool for students (Ji et al., 2025). Built on large language models, GenAI supports personalized and self-directed learning through functions like information retrieval, content generation, and conversational interaction. However, the adoption of this emerging technology has also introduced cognitive barriers. GenAI relies on an extremely large knowledge base (OpenAI et al., 2023), and its output is highly stochastic; therefore, different prompts have a significant impact on the quality of generated responses (White et al., 2023). Thus, disparities in students’ GenAI usage competence may influence their perceived learning outcomes.

Currently, no consensus has been reached regarding GenAI’s impact on learning, as existing research presents two primary perspectives: positive and cautious. Scholars with a positive stance argue that GenAI significantly enhances learning through multiple pathways (Yakubu et al., 2025), with extensive applications in coursework and research activities (Xiao et al., 2025). On the one hand, GenAI influences students’ learning outcomes directly by offering innovative approaches to problem solving and task completion (M. Kim et al., 2025). For instance, in foreign language writing (C. Wang & Wang, 2025) or code generation (Güner & Er, 2025), students can obtain AI-generated content through interaction with AI and use it as reference material or as a final output. They can acquire procedural knowledge, like writing techniques, problem-solving strategies, and thereby improve their learning performance. Students’ positive attitudes, satisfaction, and experiences accumulated while using GenAI play a positive role in these processes (Carmi, 2025). Students’ habits, performance expectations, social impact, and personal creativity are key factors driving their adoption of GenAI (Sergeeva et al., 2025; Lin & Jiang, 2025). In contrast, GenAI influences learning outcomes through indirect pathways. At the level of students’ internal cognitive and motivational factors, GenAI positively affects learning outcomes by boosting students’ academic self-efficacy, creative self-efficacy, and learning motivation, while creative thinking plays a moderating role in this process (Y. Bai & Wang, 2025). In terms of the learning environment and emotional support, GenAI helps build emotionally supportive learning contexts through personalized feedback, adaptive tutoring and interactive design to release learners’ language learning anxiety, strengthen their emotional involvement and learning initiative, and consequently achieve better learning outcomes (Kohnke & Moorhouse, 2025).

However, some studies have presented contrasting views. They found that students expressed concerns about academic integrity, over-reliance on AI, and data privacy and security (Maxwell et al., 2025). Additionally, the use of GenAI may lead to cognitive offloading and mental laziness among students, thereby impairing their ability to solve problems independently and think critically (C. Li et al., 2026; Choudhuri et al., 2026). For instance, the use of GenAI in writing tasks enhances the fluency of text while reducing students’ understanding of the underlying structure and their ability to reason independently (Rahman et al., 2025). However, research indicates that the impact of GenAI on learning motivation is not uniformly positive. GenAI tools lack a mechanism to proactively flag the uncertainty of their outputs, which may cause students to overestimate their own capabilities, slip into passive learning patterns, and experience a gradual decline in curiosity and critical thinking, ultimately undermining their intrinsic motivation (Abdelghani et al., 2023). For students with low self-efficacy, leveraging GenAI to complete complex tasks without engaging in independent thinking or knowledge organization processes yields relatively favorable short-term learning outcomes. However, their performance deteriorates significantly in AI-unassisted contexts. Concurrently, their long-term knowledge transfer capacity is impaired, suggesting that GenAI usage may give rise to the risk of adaptive dependency (S. Li et al., 2025).

In summary, the impact of GenAI on learning is not unidirectionally positive or negative; instead, it is highly dependent on usage contexts, subject domains, individual differences, specific task types, and human–AI interaction modes. Existing studies examining the impact of GenAI on perceived learning outcomes often rely on traditional scales that measure AI-related knowledge, attitudes, or perceptions (Ng et al., 2021). However, these scales hardly capture GenAI’s unique features, such as interactive generativity and human–AI collaboration (Feuerriegel et al., 2024), and thus have limitations in the accuracy of perceived learning outcome assessment. Most research uses undergraduate students as participants, yet overlooks higher vocational students. To address this gap, targeting vocational college students as the research population, this study explores the correlations between generative AI usage behaviors and vocational students’ perceived learning outcomes, and further analyzes the potential linkage mechanisms underlying such relationships.

## 3. Methodology

**Figure 1:**
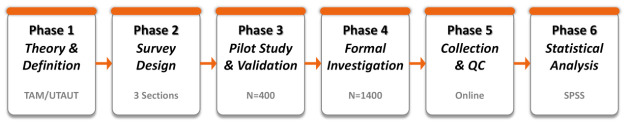
Research Design Flowchart.

This section describes the research design, instrument development, and data collection procedures. It primarily encompasses research design, development of measurement instruments, and data collection. The research design flowchart is shown in Figure 1.

### 3.1. Research Design

The questionnaire survey is a quantitative method used to explore behavioral patterns and latent relationships among variables. Its process includes administering structured questionnaires and analyzing data using multivariate statistical techniques. Through standardized items and response options, it transforms abstract research questions into quantifiable indicators, enabling the conversion of subjective individual information into analyzable data. This method was adopted to collect data on GenAI usage behavior and perceived learning outcomes from higher vocational students. Examining the relationship between these two constructs is one of the targets of this study.

The study comprised six procedures. First, (1) the core meanings and dimensional structures of the research constructs were clarified. Through a systematic review of relevant literature and theories, the operational meanings of two core variables (including GenAI usage behavior and learning outcomes) were established by integrating Technology Acceptance Model (TAM; Davis et al., 1989), Unified Theory of Acceptance and Use of Technology (UTAUT; Venkatesh et al., 2003), and existing AI literacy assessment frameworks. Second, (2) the questionnaire was designed (Appendix A). Based on the dimensional division of the core variables, the questionnaire covered basic demographic information, the GenAI usage behavior scale and the perceived learning outcomes scale. Third, (3) a pilot study was conducted and reliability and validity were assessed. Prior to the formal survey, students from a vocational college were selected for a small-scale pilot survey, with a planned distribution of 400 questionnaires. The collected questionnaire data were subjected to item analysis as well as reliability and validity tests. Based on the findings of the pilot study, the questionnaire was revised to develop the formal survey instrument. Fourth, (4) the sample size confirmation and formal investigation implementation. In scale research, the general statistical criterion suggests that the sample size should be five to ten times the number of scale items. In this study, there were 26 core measurement items. Considering invalid responses and the representativeness of samples from different majors, a total of 1400 questionnaires were distributed to ensure adequate statistical power for further empirical analysis. Fifth, (5) data collection and quality control. All questionnaires were distributed online via professional research platforms. After data retrieval, unqualified and invalid responses were excluded to maintain high overall data quality. Finally, (6) a systematic statistical analysis was conducted. Valid data were processed and analyzed using IBM SPSS Statistics (v27). Descriptive statistics were used to summarize the current status of higher vocational students’ GenAI application and learning outcomes. Differential, correlation and regression analyses were also adopted to explore the internal relationship between students’ GenAI usage behavior and their perceived learning outcomes.

### 3.2. Development of Measurement Instruments

#### 3.2.1. Development of the GenAI Usage Behavior Scale

Most existing AI literacy assessment tools measure general AI literacy and focus on technical knowledge, awareness, and ethics dimensions (Long & Magerko, 2020; Kong et al., 2021). However, as a specialized subset of AI literacy, GenAI literacy lacks targeted assessment tools. In studies assessing GenAI usage behavior, there are two categories, namely, subjective assessment scales and objective testing scales, in which subjective scales are more prevalent.

Zhang et al. (2025) referenced mature human–computer interaction frameworks, the motivation-oriented framework and the interaction-oriented model to develop the GenAI Engagement Scale. This scale takes usage frequency and interaction style as its core dimensions. The usage frequency dimension measures how often users use GenAI for self-interest-oriented and task-oriented purposes. The interaction style dimension assesses users’ interaction methods with GenAI across three sub-dimensions: exploratory, expressive, and precision-focused. Lee et al. (2026) integrated and expanded the EU’s DigComp and other existing frameworks. They added specialized GenAI skills indicators and finally proposed GenAIComp. This scale consists of five core components: information and data literacy, communication and collaboration, digital content creation, safety and ethics, and problem-solving.

In research on objective assessment scales, Jin et al. (2025) developed the GenAI Literacy Assessment Test based on Classical Test Theory and Item Response Theory. They developed a 20-item multiple-choice test covering knowledge and understanding, use and application, evaluation and creation, and ethics. Markus et al. (2025) developed and validated the AI Competency Objective Scale, thereby addressing limitations in existing AI literacy assessments, e.g., over-reliance on subjective self-reports and the absence of generative AI dimensions. This scale integrated items with high content validity from established scales and incorporated a GenAI literacy dimension. The final multiple-choice scale covered AI application, AI creation, AI identification, AI ethics, GenAI, and AI understanding.

Existing studies have attempted to construct assessment dimensions for GenAI usage behavior from multiple perspectives. Subjective scales focus on usage frequency, interaction behavior, and multi-dimensional competency frameworks, while objective scales emphasize knowledge mastery, practical application abilities, and verifiable operational skills, using standardized test items for quantifiable competency assessment. However, existing tools have limitations. Most subjective assessments focus on general GenAI literacy or macro-level dimensions like interaction style and neglect users’ actual usage behavior in daily learning and life, leading to insufficient assessment of real-world usage dynamics. Moreover, existing measurement tools often suffer from vague definitions of GenAI usage habits, failing to capture critical interactive behaviors such as adjusting prompts, comparing outputs across different AI tools, and verifying response authenticity—indicators that are nonetheless essential for assessing whether users employ GenAI in a systematic and effective manner. Most studies assume GenAI usage is applicable across multiple contexts, but do not include diverse usage contexts as an independent dimension. Nevertheless, the breadth of usage contexts influences the transferability and adaptability of GenAI usage behavior.

TAM and UTAUT are classic theoretical frameworks that explain why users adopt and how they use information systems and have been widely validated and applied in the field of educational technology (Daruwala, 2026). The aforementioned two classical theoretical frameworks, along with existing AI literacy assessment frameworks were adopted to conduct a systematic analysis of GenAI usage behavior among higher vocational students, proposing an evaluation framework for such behavior comprising three dimensions: usage frequency, habits, and contexts (Table 1). In usage frequency, based on the core logic that perceived usefulness in TAM drives the intensity of usage behavior and drawing on existing measurement approaches, two additional items (degree of behavioral dependence and task-oriented frequency) are incorporated. The overall focus is on assessing the frequency and dependency with which vocational college students use GenAI in their academic and daily lives. In usage habits, drawing primarily on the procedural competencies in the AI literacy framework, the focus is on assessing users’ interaction optimization behavior, information screening behavior, and critical adoption behavior during dynamic interactions with generative artificial intelligence. This aims to measure whether higher vocational students possess practical abilities to use GenAI efficiently and critically. In usage contexts, the construct of facilitating conditions from UTAUT is introduced as the primary theoretical basis. In UTAUT, facilitating conditions specifically measure the availability of resources, technical support, and system compatibility in the environment where users are situated, and they are essentially the core variable of this theory for capturing context differences. Given the characteristic of practical teaching for higher vocational students being integrated throughout the entire talent development process, this dimension divides usage contexts into three parts: learning, living, and developmental contexts, aiming to examine differences in usage behavior under varying levels of convenience and assess the extent to which students use GenAI across different situations.

#### 3.2.2. Development of the Perceived Learning Outcome Scale for Higher Vocational Students

Internationally, the classification of educational objectives is equivalent to that of learning outcomes. Accordingly, relevant research on learning outcome classification is mainly based on in-depth analysis of educational objective classification systems. American educator Bloom (1956) and his colleagues put forward Bloom’s Taxonomy of Educational Objectives, dividing learning outcomes into three major domains: cognition, affection and psychomotor skills (Krathwohl et al., 1964; Simpson, 1972). Nevertheless, such classification standards are mainly designed for general education, which focus on cognitive process division and define objectives from the perspective of learning procedures.

On the contrary, American psychologist Gagné (1985) categorized students’ learning outcomes into five acquired competencies, namely, verbal information, intellectual skills, cognitive strategies, psychomotor skills and attitudes, according to differences in internal psychological mechanisms and external behavioral performances. From the perspective of cognitive psychology, he also systematically elaborated the internal and external learning conditions required for each type of learning outcome.

At present, there is no unified and widely accepted classification standard for vocational education objectives within the vocational education research field. The core orientation of vocational education lies in cultivating students’ vocational competencies. German vocational education has long been acknowledged as a mature and effective model, and its dual vocational training system aims to develop students’ vocational action competence, which consists of professional competence, methodological competence and social competence. This competency system not only covers the ability to finish specific professional tasks, but also involves the overall grasp of complete work procedures involving production, technology, labor organization and social communication, realizing the integration of theoretical knowledge and practical experience (Frey & Ruppert, 2017). In China, mainstream vocational education objective classification generally follows the three-dimensional framework of knowledge, skills and literacy, yet this framework still has obvious deficiencies in adapting to complex vocational contexts and cultivating students’ sustainable development capabilities.

Based on the core framework encompassing knowledge, skills and comprehensive literacy, this study takes into account the characteristics of vocational education, the learning trajectories of higher vocational students, and their competency requirements for future careers, and further categorizes higher vocational students’ perceived learning outcomes into three dimensions: knowledge mastery, skill application, and competency development (Table 2). The knowledge mastery dimension mainly reflects students’ mastery of professional core theories and concepts, which can effectively measure their basic professional cognitive level. The skill application dimension embodies the practical nature of vocational education, focusing on evaluating students’ ability to conduct standard professional practical operations and solve typical professional problems, so as to reflect their practical vocational operational proficiency. The competency development dimension emphasizes comprehensive abilities formed in the learning process including communication competence, time management ability and independent problem-solving capacity, which are used to assess students’ sustainable development potential and adaptive capacity to complex workplace environments.

Drawing on relevant research on GenAI usage behavior and perceived learning outcomes, research hypotheses for the present study are proposed as follows:

**H1.**  *Usage frequency has a significant positive predictive effect on perceived learning outcomes.*

**H2.**  *Usage habits have a significant positive predictive effect on perceived learning outcomes.*

**H3.**  *Usage contexts have a significant positive predictive effect on perceived learning outcomes.*

**H4.**  *Among all dimensions of GenAI usage behavior, usage habits exert the strongest positive predictive effect on perceived learning outcomes.*

#### 3.2.3. Pre-Survey and Findings of Reliability and Validity Assessments

Prior to the formal distribution of the questionnaire, item analysis and tests of reliability and validity are required to ensure the scientific rigor, reliability, and validity of the questionnaire. In the pre-survey phase, 400 questionnaires were distributed, with a total of 329 valid responses collected. The GenAI Usage Behavior Scale and the Higher Vocational Students’ Perceived Learning Outcomes Scale consist of 15 and 11 items, respectively, both using a 5-point Likert scale (1 = Strongly Disagree, 2 = Disagree, 3 = Neutral, 4 = Agree, 5 = Strongly Agree). Item analysis, validity analysis, and reliability analysis were conducted separately for the two scales. In terms of item analysis, the critical ratio method, namely the extreme group comparison method, was adopted to examine the discrimination ability of each measurement item. The specific procedures are presented as follows. First, calculate the total score of each respondent. Second, divide all samples into high-score group and low-score group according to the 27% and 73% percentile critical values. Third, conduct an independent-samples *t*-test to compare the item score differences between the two groups. Fourth, revise or delete items with insignificant group differences (*p* > 0.05). The results showed that all 26 items had good discrimination effect with *p* < 0.05.

In addition to critical ratio screening, homogeneity analysis was also employed for item selection. A higher Pearson correlation coefficient between a single item and its dimension total score represents better internal consistency and higher consistency with the latent variable to be measured. Items with correlation coefficients lower than 0.4 were regarded as poorly fitted and ought to be removed. In this study, the correlation coefficient of all items with their corresponding dimensions was above 0.4 and reached statistical significance (*p* < 0.05), which proved that all items were highly consistent with the dimensional constructs and no items needed to be eliminated.

Thirdly, validity analysis was further conducted to verify the reliability and accuracy of the questionnaire. For the Generative AI Usage Scale, the KMO value reached 0.949, exceeding the threshold of 0.7, and the *p*-value of Bartlett’s Test of Sphericity was less than 0.001, which demonstrated that the scale items possessed satisfactory construct validity and were eligible for factor analysis. Principal Axis Factoring was adopted for factor extraction, followed by Promax Rotation to perform exploratory factor analysis (EFA) on all scale items.

According to the factor loading results, Item C1 exhibited severe cross-loading. Its loading on the primary Factor 3 was 0.441, while the cross-loading on Factor 1 reached 0.415, with a mere difference of 0.026 between the two values—far below the 0.20 criterion for discriminant validity. Consequently, Item C1 was eliminated.

After removing Item C1, EFA was rerun, yielding a KMO value of 0.942 (*p* < 0.001). Based on the criterion of eigenvalues greater than one and the inflection point on the scree plot, three common factors were retained. These three factors jointly explained 64.782% of the total common variance, exceeding the 60% benchmark recommended for social science research. The correlation matrix after rotation showed inter-factor correlation coefficients ranging from 0.539 to 0.675, all staying below the discriminant validity cutoff, which indicated that the three dimensions were interrelated yet conceptually distinct. As shown in the Pattern Matrix, all retained items attained favorable loadings on their respective target factors: Factor 1 consisted of Items B1–B5 with loadings from 0.791 to 0.881; Factor 2 contained Items A1–A5 with loadings ranging from 0.563 to 0.958; Factor 3 covered Items C2–C6 with loadings between 0.417 and 0.795. All retained items had primary loadings above 0.40 without substantial cross-loadings, confirming the scale’s distinct three-factor structure and sound construct validity.

For the Higher Vocational Students’ Perceived Learning Outcomes Scale, the KMO value was 0.961 and Bartlett’s Test of Sphericity returned a *p*-value less than 0.001, suggesting the dataset was suitable for factor analysis. The factor loading results revealed severe cross-loadings for Items I1 and I3. Item I1 had a loading of 0.272 on its theoretical primary Factor 2 but a cross-loading of 0.460 on Factor 1, generating a difference of only 0.188 that failed to meet the 0.20 discriminant validity standard and contradicted its theoretical dimension assignment, so Item I1 was deleted. Item I3 obtained a primary loading of 0.416 on Factor 2 yet a high cross-loading of 0.447 on Factor 1; the gap between the two loadings also fell short of the discriminant validity threshold and conflicted with its theoretical classification, thus Item I3 was likewise removed.

EFA was reimplemented after deleting I1 and I3, producing a KMO value of 0.949 (*p* < 0.001). Three factors were extracted in total: Factor 1 comprised Items H1–H3 with loadings spanning 0.759 to 0.881; Factor 2 included Items I2, I4, I5 and I6 with loadings from 0.483 to 0.871; Factor 3 contained Items J1–J3 with loadings ranging from 0.659 to 0.845.

Finally, internal consistency reliability was tested via Cronbach’s alpha coefficient. The overall Cronbach’s α of the GenAI usage behavior scale was 0.930, and the α values of its three sub-dimensions (usage frequency, usage habits, usage contexts) were all higher than 0.87. The overall Cronbach’s α of the perceived learning outcomes scale was 0.962, and the α coefficients of its three sub-dimensions (knowledge mastery, skill application, competency development) all exceeded 0.9. The above findings demonstrated that both formal scales and their sub-dimensions possessed excellent internal consistency, which could be applied to subsequent formal questionnaire survey and empirical data analysis.

### 3.3. Data Collection

To ensure regional coverage and institutional representativeness, 2–3 higher vocational colleges were selected from each of China’s four geographic regions (eastern, central, western, and northeastern), resulting in a final sample of 10 institutions. The questionnaire was formatted as a standardized online link; students completed self-administered surveys anonymously and independently, with a single submission per participant enforced to avoid duplicates.

The formal questionnaire comprised three core sections: personal basic information, the GenAI Usage Behavior Scale, and the Higher Vocational Students’ Perceived Learning Outcomes Scale. The personal basic information section includes 3 demographic items capturing gender, academic year, and broad major category. The 15-item GenAI Usage Behavior Scale covers three dimensions (usage frequency, usage habits, usage contexts), with each dimensional score calculated as the mean of its corresponding items; the overall GenAI usage score is the mean of the three dimensional scores, justified by equal item counts across dimensions and satisfactory internal consistency—higher scores indicate more engaged GenAI usage behavior among higher vocational students. The 10-item Perceived Learning Outcomes Scale involves three dimensions (knowledge mastery, skill application, competency development), with each dimensional score also being the mean of its respective items.

To calculate the comprehensive score of higher vocational students’ perceived learning outcomes, the Analytic Hierarchy Process (AHP) was adopted in this study to assign weights to each indicator. The rationale for selecting AHP is elaborated from both theoretical and practical perspectives.

From a theoretical perspective, the Analytic Hierarchy Process (AHP) is well-suited for weight determination in complex multi-criteria and multi-hierarchical indicator systems, which integrates qualitative expert experiential knowledge with quantitative weight estimation and conforms closely to the theoretical attributes of the multi-tiered evaluation framework adopted in this research. In contrast to objective weighting approaches including the entropy weighting method and the coefficient of variation method, the AHP possesses the merit of incorporating subjective domain expertise from specialists, thus addressing the inherent limitation of objective weighting techniques that may disregard the practical substantive importance of each indicator. When compared with the independent Delphi method, the AHP standardizes deviations in subjective judgments to a certain degree through consistency verification, leading to relatively robust estimation results. Notably, the AHP method fundamentally hinges on experts’ subjective evaluations. The derived weighting outcomes mirror the collective consensus of a specific expert panel on the relative priority of indicators at a particular temporal juncture.

Practically, AHP has been widely adopted for indicator weighting in existing empirical research on vocational learning outcome assessment and related measurement studies, such as vocational skill evaluation and comprehensive competency appraisal of students, thus establishing solid practical grounds for its use in the present work. Furthermore, the hierarchical indicator system constructed in this study takes the comprehensive level of learning outcomes as the target layer and the three core dimensions as the criterion layer, which satisfies the fundamental data requirements of the AHP technique.

To measure the comprehensive level of perceived learning outcomes, the Analytic Hierarchy Process was employed to determine indicator weights: 15 vocational education experts with over 5 years of teaching or research experience were invited to conduct pairwise comparisons of the three dimensions using Saaty’s 1–9 scale to assess their relative importance. The Consistency Ratio of all experts’ judgment matrices was less than 0.1 (indicating acceptable consistency); the geometric mean method was then used to synthesize the matrices, and normalization was performed to obtain the weight vector of each dimension. Finally, the weights of knowledge mastery, skill application, and competency development were rounded to 0.2, 0.4, and 0.4 respectively. Thus, the formula for the comprehensive perceived learning outcomes score is: Comprehensive Score = 0.2 × Knowledge Mastery Score + 0.4 × Skill Application Score + 0.4 × Competency Development Score.

During the formal questionnaire distribution phase, a total of 1400 questionnaires were distributed, and 1213 responses were retrieved. After eliminating straight-line responses with identical answers to all items, 1110 valid questionnaires were retained for subsequent analysis. In terms of gender composition, there were 388 male and 722 female participants, corresponding to a male-to-female ratio of approximately 1:2. In terms of grade distribution, first-year students accounted for the largest share at 70% of the total sample, while the number of third-year students was relatively small. This imbalance can be attributed to the fact that most third-year students participate in off-campus internships, making questionnaire collection difficult for this cohort. With regard to major categories, samples were collected from 18 out of 19 major groups, excluding only water conservancy. The Electronics and Information category contained the largest sample, accounting for 25.9% of all valid responses, followed by Medicine & Health and Culture & Arts. The remaining major categories had comparable sample sizes with no obvious disparities.

## 4. Results

The core of empirical research is to reveal causal relationships among phenomena. Definite conclusions can only be drawn through scientific data analysis. Researchers can transfer the static data into meaningful insights through systematic processing, thereby interpreting and validating educational phenomena. IBM SPSS (v27) was used to perform descriptive, difference, correlation, and regression analyses on the valid responses (The survey data can be found in the Supplementary File). The two aims of this chapter are to examine the current status of GenAI usage behavior and perceived learning outcomes among higher vocational college students and explore the internal relationship between GenAI usage and students’ perceived learning outcomes. In addition, to avoid potential common method bias (CMB) during measurement, Harman’s single-factor test was employed for diagnosis: all measurement items were subjected to unrotated exploratory factor analysis, and CMB was assessed by examining whether the first factor accounted for most of the variance. The analytical results indicated that the first extracted factor accounted for 43.12% of the total variance, which fell below the 50% cutoff criterion. In addition, the exploratory factor analysis did not yield a single factor; items loaded distinctly onto six theoretical constructs, corresponding to the three dimensions of each scale.

### 4.1. Descriptive Statistical Analysis

To examine the current status of GenAI usage behavior and perceived learning outcomes among higher vocational students, descriptive statistics were conducted on both the overall status and its sub-dimensions (Table 3).

For GenAI usage behavior, the overall mean score was 3.75 (SD = 0.67) indicating a moderately high level of engagement. The relatively small standard deviation reflects low data dispersion, with scores concentrated around the mean, suggesting that most students have acquired basic GenAI usage competencies. Among the sub-dimensions, usage frequency had the lowest mean (M = 3.59, SD = 0.86) and the largest standard deviation, indicating overall low GenAI usage frequency and significant individual differences in usage frequency. Usage habits had the highest mean (M = 3.95, SD = 0.74), implying that most students use GenAI in a relatively rational and scientific manner while maintaining critical thinking. Usage contexts had a mean of 3.69 (SD = 0.78), reflecting students’ widespread GenAI practices across both academic and daily contexts. Among these, applying GenAI to learn new skills of interest is relatively more common.

For perceived learning outcomes, the overall mean score was 4.01 (SD = 0.57), indicating that students currently have a relatively good grasp of knowledge, skills, and personal development competencies. The small standard deviation suggests a concentrated score distribution, implying that most students achieve above-average perceived learning outcomes. Among the sub-dimensions, knowledge mastery had the lowest mean (M = 3.95, SD = 0.64) and a higher standard deviation, indicating deficiency in professional knowledge acquisition and significant individual differences. Skill application matched the overall mean (M = 4.01, SD = 0.59), reflecting high average proficiency in practical operations, skill transfer to real-world work contexts, and problem-solving, as well as a relatively large proportion of highly skilled students. Competency development had the highest mean (M = 4.03, SD = 0.60), indicating strong performance in idea expression, time management, and independent problem analysis, with high group consistency.

### 4.2. Differential Analysis

Differential analysis can be adopted to detect whether samples with distinct demographic background characteristics demonstrate statistically significant disparities across core variables, thereby offering empirical references for an in-depth interpretation of the linkage mechanisms between generative AI usage behaviors and perceived learning outcomes. The extent of variation in perceived learning outcomes across gender, grade level, and major category is examined, with the results presented in Table 4.

First, an independent samples *t*-test was conducted to compare differences in perceived learning outcomes between different genders. The null hypothesis assumed that the population means of learning outcomes across all sub-dimensions were equal for male and female students. The alternative hypothesis assumed that population means differed between the two groups. Test results were *p* > 0.05, indicating no statistically significant differences between male and female students in knowledge mastery, skill application, competency development, or overall perceived learning outcomes. In other words, gender is not a critical factor in learning outcomes among higher vocational students.

Second, one-way analysis of variance (one-way ANOVA) was adopted to examine differences across grades in all dimensions of vocational students’ perceived learning outcomes. The null hypothesis assumed equal population means of each perceived learning outcome dimension across all grades, whereas the alternative hypothesis posited unequal population means for at least two grade groups. The analytical results revealed that the mean scores of knowledge mastery, competency development and the overall perceived learning outcome showed no statistically significant discrepancies among different grades (*p* > 0.05). Only the skill application dimension yielded a *p*-value below 0.05, indicating significant inter-grade differences. Further post hoc multiple comparisons via the LSD test demonstrated that first-year students obtained significantly higher skill application scores than second-year students (*p* = 0.02 < 0.05). This disparity may stem from the varying difficulty levels of skill assessments assigned to each grade: first-year training focuses on fundamental operational tasks, while second-year coursework involves more sophisticated practical operations, thereby creating observable gaps in this specific dimension.

Third, ANOVA was also conducted to compare perceived learning outcomes across major categories. The null hypothesis assumed equal population means of learning outcomes across all major categories, while the alternative hypothesis stated that at least two major categories had unequal population means. The result was *p* < 0.001, indicating significant differences in both sub-dimensions and overall perceived learning outcomes across major categories. Specifically, students in Education & Physical Education, Food, Pharmaceuticals & Grains, and Transportation categories obtained higher scores in professional knowledge mastery, skill application, and competency development, whereas students in Civil Engineering & Architecture, Biology & Chemical Engineering, and Electronics & Information Technology categories scored relatively lower. These disparities may stem from differences in the knowledge systems and academic difficulty of the respective curricula.

### 4.3. Correlation and Regression Analysis

To thoroughly unpack the intrinsic correlations between each dimension of vocational students’ generative AI usage behaviors and their perceived learning outcomes, this study first conducts Pearson product-moment correlation analysis on all dimensional variables to identify potential correlational relationships among constructs. Building upon the correlation results, multiple linear regression analysis is further employed to examine the predictive associations between the multi-dimensional generative AI usage behaviors and vocational students’ perceived learning outcomes, as well as to compare the relative predictive contribution of each dimension.

#### 4.3.1. Correlation Analysis

Regarding the correlations among the GenAI usage sub-dimensions (Table 5), there were significant positive correlations (*p* < 0.01) among those sub-dimensions, and overall usage behavior. Usage contexts and overall usage behavior share the largest correlation coefficient. In other words, the sub-dimensions of vocational students’ GenAI usage behavior exhibit high internal consistency. Specifically, usage contexts and overall usage behavior are most closely related.

In terms of the correlations among the perceived learning outcomes sub-dimensions, there are significant positive correlations among knowledge mastery, skill application, competency development, and overall perceived learning outcome (*p* < 0.01). Skill application has the strongest correlation with overall perceived learning outcomes, which corresponds to higher vocational education’s core mission of cultivating skilled talents.

For the correlations between GenAI usage sub-dimensions and perceived learning outcome sub-dimensions, GenAI usage behavior exhibits significant positive correlations with perceived learning outcomes. The correlation coefficients range from 0.106 to 0.418, which indicates that the two sets of sub-dimensions are interrelated, however, they have not yet reached the level of high coupling. Among usage dimensions, usage habits exhibit the strongest correlation with perceived learning outcomes.

#### 4.3.2. Regression Analysis

Correlation analysis can identify the correlation magnitude and direction between two variables, while failing to recognize the mechanism through which GenAI usage behavior sub-dimensions influence learning outcomes and their relative importance. However, multiple linear regression analysis can address these limitations. GenAI usage frequency, habits, and contexts among higher vocational students were used as independent variables; gender and grade were incorporated as two demographic control variables, with perceived learning outcomes as the dependent variable; the regression results were presented in Table 6.

The F-test (F = 51.347, *p* < 0.001) confirms the overall validity of the regression model. The basic multiple regression framework is $Y=\beta_{0}+\beta_{1}X_{1}+\beta_{2}X_{2}+\beta_{3}X_{3}+\varepsilon$ (where ε denotes the error term). Substituting the data in Table 6 yields the regression equation for the relationship between higher vocational students’ GenAI usage behavior and their perceived learning outcomes.

*Y* = 2.72 − 0.122*X*_1_ + 0.258*X*_2_ + 0.196*X*_3_ − 0.059*Z*_1_ − 0.028*Z*_2_ − 0.023*Z*_3_

In this equation, Y represents perceived learning outcomes, X_1_ represents GenAI usage frequency, X_2_ represents GenAI usage habits, and X_3_ represents GenAI usage contexts. Z_1_ is a grade dummy variable coded to indicate second-year enrollment (1 = second-year student, 0 = otherwise); Z_2_ is another grade dummy variable representing third-year status (1 = third-year student, 0 = otherwise); Z_3_ is a gender dummy variable denoting male respondents (1 = male, 0 = female). When other variables are controlled for, for each one-unit increase in GenAI usage frequency, the statistically associated change in perceived learning outcomes is a decrease of 0.122 units; for each one-unit increase in GenAI usage habits, the statistically associated change in perceived learning outcomes is an increase of 0.258 units; for each one-unit increase in GenAI usage contexts, the statistically associated change in perceived learning outcomes is an increase of 0.196 units.

In terms of the direction of association, there is a significant negative statistical correlation between GenAI usage frequency and higher vocational students’ perceived learning outcomes, which contradicts the positive predictive direction hypothesized in H1; thus, H1 is not supported. However, this negative coefficient does not indicate that usage frequency itself has an adverse impact on perceived learning outcomes. Its emergence may be explained by specific behaviors among higher vocational students: higher usage frequency is often accompanied by problematic learning practices, such as random use without clear learning goals, over-reliance on generated content, and a lack of active processing or in-depth thinking. These behaviors make it difficult to translate AI usage into positive learning gains, ultimately leading to the observed negative statistical association. In contrast, both usage habits and contexts exhibit a significant positive predictive effect on perceived learning outcomes, so hypotheses H2 and H3 are supported. The results indicate that in the process of generative AI-assisted learning, “how to use” and “in what contexts to use” show a positive statistical association with perceived learning outcomes. In terms of association strength, the statistical association between usage habits and learning outcomes is the strongest, followed by usage contexts, while that of usage frequency is relatively weak. This aligns with the expectation in hypothesis H4 that usage habits have the strongest positive predictive effect, so H4 is supported.

## 5. Discussion

The potential impacts of GenAI usage on perceived learning outcomes were explored by analyzing the relationship between the two variables and developing two context-specific assessment scales. The GenAI Usage Behavior Scale integrates TAM (Davis et al., 1989), UTAUT (Venkatesh et al., 2003), and existing AI literacy assessment frameworks to more precisely measure students’ actual usage patterns across frequency, habits, and contexts.

The Perceived Learning Outcomes Scale draws on the talent development objectives of vocational education and the practical demands for practical orientation, job adaptability, and sustainable development. Perceived learning outcomes are divided into knowledge mastery, skill application, and competency development. Both scales demonstrate strong contextual relevance and target validity, offering new measurement perspectives for assessing GenAI usage behavior and perceived learning outcomes. The foundation for analyzing their interrelationships is also established.

First, this study enriches the analytical perspective on GenAI usage behavior. Most existing research on GenAI usage has focused on tertiary students without distinguishing undergraduates from higher vocational students, and samples have been dominated by the former. This approach overlooks contextual differences in emerging technology engagement across educational tracks. Compared to undergraduate education, higher vocational education places greater emphasis on practical orientation, job adaptability, and professional competency development. Therefore, the distinctive characteristics of vocational education and the core technical features of GenAI were integrated to characterize students’ GenAI usage behavior in terms of frequency, habits, and contexts. Unlike a single-dimension measure of AI use versus non-use, the scale emphasizes strategic, critical, and context-specific usage behavior, providing a measurement framework for future assessments.

Second, distinct patterns in perceived learning outcomes across student background characteristics were revealed. There is no significant difference in knowledge mastery, skill application, competency development, or overall perceived learning outcomes between male and female students. In other words, gender is not a critical factor influencing perceived learning outcomes. The differences in learning outcomes were more likely attributable to variations in learning engagement, specialized training, and technology usage patterns. In terms of grade differences, significant disparities across vocational students from different academic years were only detected in the skill application dimension of perceived learning outcomes. Post-hoc comparisons further revealed that first-year students obtained higher scores than second-year students. Such discrepancies may be attributable to varying difficulty levels of curricular assignments across grades, as learners tend to produce less accurate self-assessments when confronted with more complex tasks. Given that upper-grade students are generally assigned more sophisticated practical coursework and skill requirements, their self-reported proficiency is likely to be relatively lower. This finding should not be simply interpreted as evidence that junior students possess superior actual skills compared to senior cohorts. Systematic differences in curriculum content, knowledge domains, teaching practices, and career orientations across majors shape students’ cognitive approaches, and academic background moderates the relationship between learning motivation and academic achievement (Breetzke et al., 2024). In other words, learning outcomes are highly associated with majors, with notable disparities across sub-dimensions. Thus, taking major background into consideration is essential when examining GenAI’s impact on perceived learning outcomes.

Third, a significant positive correlation was identified between GenAI usage behavior and perceived learning outcomes, with usage habits exhibiting the strongest correlation with perceived learning outcomes. Metacognitive habits, such as active monitoring and deep reflection, act as critical mediating variables in translating AI-assisted learning into real and transferable perceived learning outcomes. Over-reliance on GenAI for task completion may weaken long-term learning capacity (Fan et al., 2025). In fact, there exists an intimate correlation between AI usage habits and academic integrity. Students with sound generative AI usage habits are more inclined to question, verify, and critically examine AI-generated outputs, which enables them to maintain heightened awareness and self-discipline in compliance with academic norms when citing or adopting AI-produced content. In contrast, students lacking healthy usage habits are prone to uncritically adopt outputs generated by AI. Such habitual over-reliance may inadvertently lead to plagiarism and other forms of academic misconduct. In the absence of clear institutional regulations governing AI utilization, this ambiguous grey area of AI application tends to become prevalent. It not only intensifies threats to the academic integrity framework but also diminishes the diagnostic value of assessments for evaluating students’ genuine competencies. In other words, academic integrity and metacognitive habits are closely intertwined. Sound generative AI usage habits encompass not only technical competencies such as optimized human–AI interaction and information screening, but also vigilance over the legitimate boundaries of AI content as well as sound ethical judgment. Thus, vocational institutions should pay more attention to cultivating effective usage habits rather than merely expanding GenAI access in educational practices.

Finally, the regression predictive effects of different dimensions of GenAI usage behavior on the perceived learning outcomes of vocational college students were validated. In the regression model, GenAI usage frequency has a significant negative predictive effect on higher vocational students’ perceived learning outcomes, while both usage habits and usage contexts have significant positive predictive effects on perceived learning outcomes. On one hand, the predictive effect of GenAI usage frequency on the perceived learning outcomes of higher vocational students does not follow the logic that more frequent usage leads to better perceived learning outcomes. Over-reliance on GenAI may enhance task efficiency but undermine independent thinking and knowledge construction, thereby potentially hindering the improvement of perceived learning outcomes (Lepp & Kaimre, 2025). Thus, intentional habits and diverse contexts are beneficial to perceived learning outcomes. On the other hand, the positive predictive effect of usage habits on perceived learning outcomes is the most pronounced, which is consistent with the results of the correlation analysis in this study. Structured usage can improve students’ perceived learning outcomes by enhancing their competencies in problem-solving, critical thinking and communication (Hassan, 2026). These results deepen understanding of the mechanisms of GenAI usage among higher vocational students and provide theoretical guidance for AI integration in education.

As GenAI gradually integrates into students’ daily learning lives, its application scope in the field of education has become increasingly extensive. As an important intelligent tool to assist students’ learning, GenAI can enhance students’ perceived learning outcomes through multiple pathways. However, the effect of GenAI on learning is not automatically achieved by the technical tool itself; rather, it depends on students’ usage behavior. Based on these findings, to promote effective GenAI usage behavior and improve perceived learning outcomes, colleges, teachers, and students should each have clear priorities and collaborate synergistically.

At the school level, the focus should shift from “encouraging usage” to “context standardization and habit cultivation”. GenAI usage contexts have a significant positive predictive impact on perceived learning outcomes, as evidenced by the findings of this study. Therefore, schools should not merely stay at the vague advocacy level of encouraging students to use AI, but instead formulate clear behavioral guidelines for different learning contexts. Through context-based standardization, schools can prevent students from misusing AI in inappropriate contexts, thereby amplifying the positive effect of usage contexts on perceived learning outcomes. Given the significant differences in learning outcomes across different major categories, schools should avoid homogeneous AI application promotion strategies. For knowledge-intensive majors, the focus can be on providing application support for AI in knowledge integration and expansion. For skill-operation majors, the emphasis should be on developing AI’s auxiliary functions in contexts such as operation process simulation and case analysis, while supporting the development of major-specific prompt libraries and application templates, and integrating AI literacy into the curriculum (Hershkovitz et al., 2025).

At the teacher level, the focus should shift from “tool demonstration” to “systematic guidance on usage habits”. Given the positive statistical correlation between usage habits and perceived learning outcomes, as guides for students’ learning, professional teachers should help students understand the working principles, capabilities, and limitations of GenAI, and cultivate students’ critical thinking and ethical values (Bower et al., 2024). They should focus on key usage behavior such as question optimization, content verification, information screening, result comparison, and tool selection. Combining the practice-oriented characteristics of higher vocational education, teachers should systematically design relevant teaching content using practical application contexts of professional courses as teaching cases (K. Wang et al., 2024). Meanwhile, to enhance transparency, teachers should clearly specify the scope of AI usage, permitted and prohibited situations, and corresponding academic integrity requirements in the course syllabus (Dabis & Csáki, 2024).

At the student level, the shift should be made from “being able to use the tool” to “being proficient in using the tool”. As the direct users of GenAI, students should continuously enhance their digital literacy, recognize that simply increasing the frequency of AI usage may instead have an adverse impact on perceived learning outcomes, and avoid the phenomenon of cognitive offloading caused by over-reliance on GenAI for information retrieval and decision-making (Gerlich, 2025). Meanwhile, students should improve their ability to transfer and apply GenAI across multiple learning contexts and proactively explore the rational use of GenAI in different tasks such as knowledge learning, skill application, project practice, and vocational scenario simulation. Through the structured use of GenAI, students can improve their academic performance (Xu et al., 2025).

The limitations of this study are as follows. First, the measurement scales can be further refined. Both the self-developed scale for generative AI usage behaviors and the scale measuring vocational college students’ perceived learning outcomes exhibit satisfactory reliability and validity and were finalized as formal measurement instruments after revisions based on a pilot survey. Nevertheless, confirmatory factor analysis (CFA) was not adopted to cross-validate the robustness of their theoretical latent structures, which, to some extent, weakens the empirical evidence supporting the construct validity of the two scales. In terms of measurement coverage, the generative AI usage behavior scale mainly centers on three dimensions: usage frequency, usage habits and usage contexts, while touching insufficiently upon deeper AI literacy dimensions including ethical judgment, risk awareness and awareness of academic norms. In addition, the competency development dimension within the perceived learning outcomes scale contains a relatively small number of items. Therefore, further expansion and refinement of the scale items are required in follow-up investigations.

Second, weight assignment via the Analytic Hierarchy Process (AHP) carries inherent subjectivity. Although the AHP method is capable of incorporating domain experts’ professional insights and mitigating subjective biases to a moderate extent through consistency tests, the resultant weights fundamentally rest on the experiential perceptions of the expert panel. Such weights reflect the collective judgments of a specific group of specialists regarding the relative importance of indicators at a single time point and cannot be regarded as fully objective weighting values. Alternative expert panels or different weighting algorithms may yield divergent weight distributions. Accordingly, this study interprets weighted calculation results cautiously, treating the weights as quantitative reflections of aggregated expert experience within the research context rather than absolutely objective measurement benchmarks.

Third, common method bias cannot be completely eliminated. While preliminary statistical evidence from Harman’s single-factor test and corresponding theoretical reasoning are provided to address this issue, both independent and dependent variables were measured via self-reported questionnaires administered to identical participants at a single time point, leaving the potential for common method bias unaddressed. Such bias may distort the estimated associations between variables and undermine the robustness of the research findings. Future inquiries may adopt multi-source data (e.g., peer ratings, teacher evaluations or objective academic performance indicators), multi-wave cross-sectional designs or longitudinal frameworks to effectively mitigate or eliminate confounding effects induced by common method variance.

Fourth, the set of control variables incorporated into the regression model remains incomplete. Even though gender and grade were included as demographic controls, the model still suffers from notable constraints stemming from data accessibility and the original research design. Several theoretically critical predictors of learning outcomes were excluded from the model, such as students’ prior academic achievement, socioeconomic status, artificial intelligence literacy, digital literacy, learning motivation, academic self-efficacy, learning engagement, teacher support and institutional-level contextual factors. The omission of these covariates may reduce the explanatory power of the regression model and introduce biases into parameter estimates. Subsequent research should embed the aforementioned variables into questionnaire designs in advance and adopt more comprehensive model specifications to delineate the relationships between generative AI usage behaviors and perceived learning outcomes with greater precision.

The future research directions are as follows. First, regarding the selection of measurement tools, as the pace of technological iteration accelerates, the types of GenAI tools continue to expand, their functions and application contexts become increasingly diverse, and the connotation of their usage behavior will further evolve. Moreover, the current study’s measurement of perceived learning outcomes relies mainly on self-reported questionnaires. This method is quite subjective, and the measured learning outcomes may largely reflect students’ confidence, motivation, or social desirability rather than actual knowledge mastery or skill improvement. Future research needs to align with the latest understanding of GenAI usage behavior, continuously update and refine its components, for example, splitting “usage frequency” and “dependency level” into two independent dimensions for measurement. On this basis, more systematic, scientific, and context-adaptable assessment tools should be developed. Meanwhile, to measure students’ learning outcomes with higher accuracy, future research should actively adopt multi-source data and objective evaluation indicators. Beyond students’ self-reported assessments, additional objective metrics such as course grades, practical training scores, instructor evaluations, internship performance reviews, professional skill test results and vocational certification outcomes ought to be incorporated. Such multi-source measurement can effectively mitigate the potential confounding influence of common method bias on research conclusions and further verify the robustness of the correlational relationships identified in the present study. In addition, in the validation phase of measurement tools, future studies can use exploratory factor analysis based on the common factor model combined with oblique rotation to further test the construct validity of this study.

Secondly, in terms of model construction and covariate adjustment, the regression model adopted in this study is relatively parsimonious, incorporating only gender and academic year as control variables while omitting other factors that may exert influences on learning outcomes. Future research should elaborate on and incorporate a full set of critical covariates, including prior academic performance, socioeconomic background, artificial intelligence literacy, digital literacy, learning motivation, self-efficacy, learning engagement, teacher support, as well as institutional-level factors, so as to unpack the associations between generative AI usage and perceived learning outcomes with greater methodological rigor. In addition, given the complexity of the relationships between variables in this study, future research can use structural equation modeling for more in-depth mechanism analysis. This will better handle multiple causal relationships and test potential mediating or moderating effects.

Thirdly, regarding sample selection and representativeness, all participants recruited in this study were sourced from vocational colleges in China, which means the generalizability of the research findings to educational contexts outside China remains unexamined. Future investigations should select vocational institutions of varying regions, tiers and operational types on a broader scale to enhance sample representativeness and the external validity of the conclusions. Furthermore, cross-cultural comparative research is encouraged to test the cross-contextual stability of the associations identified in the current work.

Finally, regarding academic integrity issues in GenAI usage, the discussion in this study remains limited. Given that GenAI can be used to complete assignments, write papers, code, and finish various tasks, future research should include discussions on issues such as plagiarism, unauthorized assistance, assessment validity, and related AI policies. It should also further clarify the moral boundaries and behavioral norms for students when using GenAI. Meanwhile, the differences in perceived learning outcomes among students of different major categories suggest that future research can focus on specific professional backgrounds. It can analyze the differentiated application effects of GenAI in contexts such as knowledge learning, skill training, and career development, so as to provide more targeted data support for teaching reforms in vocational colleges.

## 6. Conclusions

In the context of GenAI’s widespread integration into student learning, guiding students to use GenAI scientifically and effectively is of great significance for vocational education. It empowers the enhancement of students’ perceived learning outcomes and supports the cultivation of high-quality technical and skilled talents. Grounded in the cultivation requirements of vocational education (i.e., practical orientation, job adaptability, and competency development), two scales with sound reliability and validity were developed and validated in this study, which focused on higher vocational students as research subjects: the GenAI Usage Behavior Scale and the Higher Vocational Students’ Perceived Learning Outcomes Scale. Their interrelationship was subsequently examined based on data from 1110 valid questionnaires. The findings are as follows. Higher vocational students’ GenAI usage behavior is generally at a moderately high level, with scores for usage habits higher than those for usage contexts and frequency. The overall evaluation for higher vocational students’ perceived learning outcomes is rated above average, with competency development ranking highest, followed by skill application and knowledge mastery. No statistically significant gender disparities were identified in higher vocational students’ overall perceived learning outcomes, while first-year students achieved significantly higher scores than second-year students on the skill application dimension. Students from different major categories exhibit significant differences in knowledge mastery, skill application, competency development, and overall perceived learning outcomes. Both sub-dimensions of the GenAI Usage Behavior Scale and the Perceived Learning Outcomes Scale demonstrated strong internal consistency. The two variables exhibited a significant positive correlation. Specifically, usage habits show the strongest correlation with perceived learning outcomes. Usage frequency shows a statistically significant negative association with perceived learning outcomes, while usage habits and usage contexts exhibit a statistically significant positive correlation. Among these, usage habits have the strongest positive predictive effect on the perceived learning outcomes of higher vocational students. Therefore, it is recommended that colleges, teachers, and students collaborate to optimize students’ GenAI usage behavior, thereby more effectively enhancing their perceived learning outcomes in knowledge mastery, skill application, and competency development.

## Figures and Tables

**Table 1:** Dimensional Division and Indicator Connotations of GenAI Usage Behavior.

Core Dimension	Constituent Elements	Description
GenAI Usage Behavior	Usage Frequency	The frequency and dependence of individuals’ GenAI usage in learning and daily life
	Usage Habits	Individuals’ interactive optimization competence in adjusting prompts, comparing AI tools, and documenting selected tools, as well as their information discrimination and integration competence in verifying answers, when using GenAI
	Usage Contexts	The breadth of individuals’ GenAI usage across diverse contexts including learning, daily life, and entertainment

**Table 2:** Dimensional Division and Indicator Connotations of Higher Vocational Students’ perceived Learning Outcomes.

Core Dimension	Constituent Elements	Description
Higher Vocational Students’ Perceived Learning Outcomes	Knowledge Mastery	The level of cognition and comprehension of core concepts and theories within their professional domain among higher vocational students
	Skill Application	The practical competence and proficiency of higher vocational students in applying the professional operational skills they have acquired to real-world work tasks
	Competency Development	The comprehensive development level of general qualities and transferable abilities of higher vocational students

**Table 3:** Descriptive Statistical Results of GenAI Usage Behavior and Perceived Learning Outcomes Among Higher Vocational Students.

Comparison Dimensions	Sub-Dimensions	N	Min	Max	Mean	SD
GenAI Usage Behavior	Usage Frequency	1110	1	5	3.60	0.85
	Usage Habits	1110	1	5	3.95	0.74
	Usage Contexts	1110	1	5	3.69	0.78
	Overall Status	1110	1	5	3.75	0.67
Perceived Learning Outcomes	Knowledge Mastery	1110	1	5	3.95	0.64
	Skill Application	1110	1	5	4.01	0.59
	Competency Development	1110	1	5	4.03	0.60
	Overall Status	1110	1	5	4.01	0.57

**Table 4:** Results of Differential Analysis on the Sub-dimensions of Higher Vocational Students’ Perceived Learning Outcomes.

Comparison Dimensions		Knowledge Mastery	Skill Application	Competency Development	Perceived Learning Outcomes
Gender	Male	3.93 ± 0.72	4.01 ± 0.66	4.03 ± 0.66	4.00 ± 0.64
	Female	3.96 ± 0.59	4.01 ± 0.55	4.03 ± 0.56	4.01 ± 0.52
	F	20.589	9.044	7.380	11.107
	*p*	0.631	0.877	0.977	0.854
Grade Level	Freshman	3.96 ± 0.63	4.04 ± 0.58	4.05 ± 0.59	4.03 ± 0.55
	Sophomore	3.92 ± 0.67	3.93 ± 0.65	3.98 ± 0.63	3.95 ± 0.61
	Junior	3.90 ± 0.63	3.96 ± 0.58	3.98 ± 0.58	3.96 ± 0.56
	F	0.903	3.182	1.421	2.092
	*p*	0.406	0.042	0.242	0.124
Major Category	Agriculture, Forestry, Animal Husbandry & Fishery	4.02 ± 0.52	4.04 ± 0.50	4.05 ± 0.52	4.04 ± 0.47
	Resources, Environment & Safety	3.89 ± 0.83	4.02 ± 0.70	3.97 ± 0.80	3.98 ± 0.76
	Energy, Power & Materials	4.18 ± 0.68	4.15 ± 0.69	4.31 ± 0.60	4.22 ± 0.60
	Civil Engineering & Architecture	3.75 ± 0.50	3.88 ± 0.63	3.83 ± 0.58	3.83 ± 0.58
	Equipment Manufacturing	4.05 ± 0.70	4.15 ± 0.57	4.17 ± 0.57	4.14 ± 0.55
	Biology & Chemical Engineering	3.86 ± 0.44	3.91 ± 0.46	3.90 ± 0.42	3.90 ± 0.39
	Light Industry & Textiles	3.87 ± 0.43	3.88 ± 0.43	3.91 ± 0.45	3.89 ± 0.38
	Food, Pharmaceuticals & Grains	4.25 ± 0.46	4.27 ± 0.50	4.44 ± 0.48	4.33 ± 0.46
	Transportation	4.31 ± 0.69	4.34 ± 0.70	4.22 ± 0.63	4.28 ± 0.65
	Electronics & Information Technology	3.80 ± 0.63	3.89 ± 0.58	3.89 ± 0.61	3.87 ± 0.57
	Medicine & Health Care	4.13 ± 0.64	4.20 ± 0.60	4.22 ± 0.62	4.19 ± 0.58
	Finance, Economics & Commerce	3.84 ± 0.65	3.97 ± 0.55	3.97 ± 0.53	3.94 ± 0.53
	Tourism	3.33 ± 1.36	3.75 ± 1.55	3.50 ± 1.48	3.57 ± 1.46
	Culture & Art	3.88 ± 0.62	3.91 ± 0.61	3.97 ± 0.60	3.93 ± 0.57
	Journalism & Communication	4.08 ± 0.72	4.10 ± 0.64	4.12 ± 0.58	4.10 ± 0.59
	Education & Physical Education	4.40 ± 0.47	4.40 ± 0.46	4.27 ± 0.49	4.35 ± 0.44
	Public Security & Justice	3.83 ± 0.52	3.91 ± 0.45	3.86 ± 0.50	3.87 ± 0.44
	Public Administration & Services	3.98 ± 0.55	4.07 ± 0.48	4.07 ± 0.49	4.05 ± 0.49
	F	3.890	3.738	4.428	4.466
	*p*	<0.001	<0.001	<0.001	<0.001

**Table 5:** Correlation Analysis Between Higher Vocational Students’ GenAI Usage Behavior and Perceived Learning Outcomes.

Variables	GenAI Usage Frequency	GenAI Usage Habits	GenAI Usage Contexts	GenAI Usage Behavior	Knowledge Mastery	Skill Application	Competency Development	Perceived Learning Outcomes
GenAI Usage Frequency	1							
GenAI Usage Habits	0.481 **	1						
GenAI Usage Contexts	0.604 **	0.637 **	1					
GenAI Usage Behavior	0.837 **	0.820 **	0.880 **	1				
Knowledge Mastery	0.160 **	0.378 **	0.355 **	0.345 **	1			
Skill Application	0.135 **	0.411 **	0.362 **	0.349 **	0.818 **	1		
Competency Development	0.106 **	0.383 **	0.337 **	0.317 **	0.779 **	0.837 **	1	
Perceived Learning Outcomes	0.137 **	0.418 **	0.373 **	0.357 **	0.895 **	0.955 **	0.947 **	1

** *p* < 0.01.

**Table 6:** Regression Analysis Results of Higher Vocational Students’ GenAI Usage Behavior on Perceived Learning Outcomes.

Model			Unstandardized Coefficients		Standardized Coefficients	t	Sig.	Collinearity Statistics	
			B	Std. Error	Beta			Tolerance	VIF
Independent variables	(Constant)		2.72	0.09		30.356	0		
	A		−0.122	0.023	−0.183	−5.368	0	0.608	1.644
	B		0.258	0.027	0.336	9.589	0	0.578	1.729
	C		0.196	0.028	0.269	6.977	0	0.475	2.103
Control variables	Grade	Grade 2	−0.059	0.04	−0.041	−1.49	0.136	0.936	1.068
		Grade 3	−0.028	0.049	−0.015	−0.562	0.574	0.965	1.036
		Grade 1	0						
	Gender	Male	−0.023	0.032	−0.019	−0.719	0.472	0.978	1.022
		Female	0						
R^2^			0.218						
Adjusted R^2^			0.214						
F			51.347						
*p*			<0.001						
Dependent Variable: Perceived Learning Outcomes									

## Data Availability

The raw data supporting the conclusions of this article will be made available by the authors upon request.

## Supplementary Materials

The following supporting information can be downloaded at: https://www.mdpi.com/article/10.3390/bs16071166/s1.

## Appendix A. Survey on Generative Artificial Intelligence Usage Behavior and Learning Outcomes of Higher Vocational Students

Dear student:

Thanks for taking the time to participate in this survey. We are the research team from Tianjin University’s project on the impact of generative artificial intelligence, such as DeepSeek, ChatGPT, and DouBao, on higher vocational students’ learning outcomes. This questionnaire aims to investigate how usage patterns of GenAI affect students’ academic performance. The survey is conducted anonymously for academic research purposes only. Your responses will not have any negative consequences, and your information will remain strictly confidential. Thank you sincerely for your support and assistance. Wishing you happiness in your life and success in your studies!

- Personal Information (Single Choice Questions)

1. Your gender:

○ Male ○ Female

2. Your grade level:

○ Freshman ○ Sophomore ○ Junior

3. Your major’s discipline:

○ Agriculture, Forestry, Animal Husbandry & Fishery

○ Resources, Environment & Safety ○ Energy, Power & Materials

○ Civil Engineering & Architecture ○ Water Conservancy ○ Equipment Manufacturing

○ Biology & Chemical Engineering ○ Light Industry & Textiles

○ Food, Pharmaceuticals & Grains ○ Transportation

○ Electronics & Information Technology ○ Medicine & Health Care

○ Finance, Economics & Commerce ○ Tourism ○ Culture & Art

○ Journalism & Communication ○ Education & Physical Education

○ Public Security & Justice ○ Public Administration & Services

II. Higher Vocational Students’ Generative Artificial Intelligence Usage Behavior Scale (Matrix Scale Questions)

A. Generative Artificial Intelligence Usage Frequency					
	Strongly Disagree	Disagree	Neutral	Agree	Strongly Agree
I use GenAI platforms such as DeepSeek-v4, ChatGPT-3.5, Ernie Bot-5.1, Kimi-2.6 and Doubao to address problems multiple times per week	○	○	○	○	○
When facing difficulties, I prefer asking GenAI to searching for materials independently.	○	○	○	○	○
I frequently use GenAI to help complete my homework and study assignments.	○	○	○	○	○
I frequently use GenAI tools during in-class learning.	○	○	○	○	○
My learning efficiency would drop significantly without GenAI tools.	○	○	○	○	○
B. Generative Artificial Intelligence Usage Habits					
I check AI-generated answers against textbooks and credible references.	○	○	○	○	○
I adjust my prompt strategies to obtain satisfying answers when using GenAI.	○	○	○	○	○
I tend to use AI-generated content for reference rather than copy it outright.	○	○	○	○	○
I ask the same question via different GenAI tools and compare differences in their responses.	○	○	○	○	○
I record commonly used GenAI tools and their application contexts, and use them to solve targeted problems.	○	○	○	○	○
C. Generative Artificial Intelligence Usage Contexts					
I use GenAI to search for information related to course content.	○	○	○	○	○
I use GenAI to assist with homework, such as thesis writing, formula calculations, and code writing.	○	○	○	○	○
I turn to GenAI for help when encountering daily challenges such as diet management, financial management, and social interaction.	○	○	○	○	○
I communicate with GenAI for entertainment when feeling bored.	○	○	○	○	○
I use GenAI to learn new skills of interest, such as photography, video editing, and painting.	○	○	○	○	○
I use GenAI to polish resumes and revise job application letters.	○	○	○	○	○

III. Current Status of Higher Vocational Students’ Learning Outcomes (Matrix Scale Questions)

H. Knowledge Mastery					
	Strongly Disagree	Disagree	Neutral	Agree	Strongly Agree
I can roughly restate the core concepts and theories of my major courses.	○	○	○	○	○
I keep myself updated with industry-related new knowledge and standards.	○	○	○	○	○
I can connect unfamiliar professional issues with relevant knowledge I have mastered.	○	○	○	○	○
I. Skill Application					
I am proficient in performing practical operations required by my major, such as using instruments and software.	○	○	○	○	○
I can solve typical professional problems following standard procedures taught by instructors.	○	○	○	○	○
I can transfer skills learned in class to real-world workplace contexts.	○	○	○	○	○
I can cooperate with classmates to finish collaborative practical tasks.	○	○	○	○	○
I can improve my proficiency in core professional skills through repeated practice.	○	○	○	○	○
I can solve new operational problems by consulting relevant materials and iteratively refining my approach.	○	○	○	○	○
J. Competency Development					
I can express my viewpoints clearly and logically.	○	○	○	○	○
I can manage my time effectively to balance study, practice, and personal responsibilities.	○	○	○	○	○
I can analyze problems independently and form my own solutions.	○	○	○	○	○
